# Going Beyond the Catch-22 of Autism Diagnosis and Research. The Moral Implications of (Not) Asking “What Is Autism?”

**DOI:** 10.3389/fpsyg.2020.529193

**Published:** 2020-11-10

**Authors:** Jo Bervoets, Kristien Hens

**Affiliations:** ^1^NeuroEpigenEthics, Department of Philosophy, University of Antwerp, Antwerp, Belgium; ^2^Brain and Cognition Unit, Department of Psychology, Katholieke Universiteit Leuven, Leuven, Belgium; ^3^Leuven Autism Research Unit, Department of Health Sciences, Katholieke Universiteit Leuven, Leuven, Belgium; ^4^Centre for Logic and Philosophy of Science, Higher Institute of Philosophy, Katholieke Universiteit Leuven, Leuven, Belgium

**Keywords:** autism, ASD, ASC, ethics, neurodiversity, interdisciplinarity, psychiatry, anomalous monism

## Abstract

Psychiatric diagnoses such as Autism Spectrum Disorder (ASD) are primarily attributed on the basis of behavioral criteria. The aim of most of the biomedical research on ASD is to uncover the underlying mechanisms that lead to or even cause pathological behavior. However, in the philosophical and sociological literature, it has been suggested that autism is also to some extent a ‘social construct’ that cannot merely be reduced to its biological explanation. We show that a one-sided adherence to either a biological or a social explanation leads to a moral dilemma, a Catch-22, for autistics and for those living with them. Such explanations close the space for self-identifying as autistic and at the same time being considered to be in good mental health. They foreclose the possibility of making sense of the lived experience of (and with) autistics. In this paper we argue that such lack of space for moral imagination inherently leads to scientific stalemate. We propose that one can only go beyond this stalemate by taking an ethical stance in theorizing, one that enables better intersubjective understanding. Only on such a view can behavior and biology be linked without either disconnecting them or reducing the one to the other.

## Introduction

*“Orr would be crazy to fly more missions and sane if he didn’t, but if he was sane, he had to fly them. If he flew them, he was crazy and didn’t have to; but if he didn’t want to, he was sane and had to.”* Joseph Heller, Catch 22 (Heller, 1961).

Discussions about autism^1^ are caught up in simultaneous stalemates. On the one hand, there is the ethical stalemate between considering autism as a disorder or claiming it as a positive identity (Mole, 2017). On the other hand, there is the ontological stalemate between concepts of autism as a pure social construct or as having an immutable essence. Does autism allow for unification under, for instance, one (or more) biological explanations, or is it just a useful clinical category expressing that certain behaviors are deemed problematic in current societal circumstances? A key question in the latter stalemate is whether or not it makes sense at all to try to answer the question: ‘What is autism?’ (Hacking, 1999). In this paper, we demonstrate that the ethical and ontological stalemates are connected, by analyzing the moral implications of (not) inquiring into the essence of autism. A theoretical choice, whether it tries to answer the ‘What is?’ question or considers it unanswerable, always has moral implications. We show that to resolve the ethical stalemate, we must start from and incorporate the experiences of actual autistics. This puts constraints on the theoretical approaches that can be fruitfully investigated, as they are not all equally open to incorporating these experiences. We, therefore, propose that to move beyond a Catch-22 inherent in current diagnostic and research conceptualizations, we are to reconceptualize autism to offer room to imagine autistic people as moral agents who are not *fully* determined by their autism, in other words as an intrinsically ambivalent social-biological *phenomenon*. Any fruitful theoretical approach to autism will be at the same time ethically sound and truly interdisciplinary. We recognize that much recent autism research is already moving in this direction, de facto integrating autistic lived experiences and insights of both the social and exact sciences. This paper can be seen as providing an explicit philosophical underpinning of this trend in identifying how the current diagnostic conceptualization of autism forms a remaining barrier to it. We come back to this toward the end of the paper after first treating in detail the more entrenched views regarding the question ‘What is autism?’.

The neurodiversity movement, which claims that autism can be a positive practical identity, is an attempt to steer clear of pathologizing autism. An intuition at the center of the neurodiversity movement is that neurological diversity is a fact that should not be identified with psychiatric problems. It is unfair to treat the neurodiversity movement as a monolith. Milton (2017) and Chapman (2020), focus on autism from a social perspective. They put the emphasis on the idea that aspects that make life challenging for autistics are not intrinsically linked to individual flaws, but to a mismatch between the individual and the environment, and a lack of support. Jaarsma and Welin (2012) while following this reasoning focus more on autism being a neutral neurological or genetic variant in so-called cases of ‘high functioning autism’ (HFA). For them, however, autism if combined with intellectual disabilities, or ‘low functioning autism’ (LFA), can be seen as inherently disordered. But this opens up the question of which lived experience counts as genuinely autistic^2^. Why would having an IQ above a certain threshold make the difference in being allowed to positively identify as autistic? Still, if the autistic voice is not to be split in this way, an account must be given about how the everyday problems that autistic individuals may experience are related to a common underlying aspect of the autistic lived experience. As Chapman (2020) argues, the challenge is to resist abandoning the over-arching concept of autism in view of “problematic findings relating to the biological underpinnings of autism,” on the latter see Happé et al. (2006) and Waterhouse and Gillberg (2014).

As the neurodiversity movement shows, it is intuitively attractive to see autism in a way that combines elements of both essentialist and constructivist approaches. However, this then begs the question of how, if at all, they *can* be combined in a scientifically fruitful way^3^.

While we do not attempt to answer the latter question here, it is clear that the researchers investigating autism meanwhile need to proceed with a working definition of autism. At this time, that definition is given in the DSM-5 (American Psychiatric Association, 2013) entry on Autism Spectrum Disorder (ASD). In this definition, two main behavioral criteria (clauses A/B) are coupled with (what is broadly construed as) an innate developmental disorder (clause C) *and* with problematic functioning (clause D)^4^. This working definition is compatible with pure essentialist and social constructivist theories but, as we will argue, conflicts with the dimension of the lived experience of the autistic person. Indeed, the conceptualization of autism per ASD in DSM-5 inevitably puts the autistic person in a Catch-22 situation. They might indeed think: “I’d be disordered if I accept to be autistic, but if I’m in good mental health I have to accept I’m not autistic.” The fact that this dilemma poses real problems for autistics is specifically evident in cases of ‘late diagnosis,’ see for instance self-reporting in *Experiences of Adults Following an Autism Diagnosis* (Hens and Langenberg, 2018). Either autistics try to adapt, only to ultimately succumb under the pressure of their coping and compensation strategies, or they accept to self-identify with autism but will then have to face the stigma and stereotype associated with a psychiatric diagnosis referring to persistent dysfunctionality. Here, before developing our argument, it is crucial to acknowledge that seeing autism only in a light of dysfunction is luckily something that is being replaced by seeing it more and more as a locus of potential strengths. This evolution, in large part enabled by the neurodiversity movement, is the reason behind the mentioned drive for positively self-identifying as autistic. It is, however, still a painstakingly slow process^5^. This paper needs to be seen in the context of removing one of the main elements of this slowness, namely the one-on-one association between autism and disorder or dysfunction. Our focus on the latter should not be taken as a denial of the positive potential of autism as this potential is, in fact, one of the key motivations of our argument.

With these introductory elements in place, we can detail the plan of our argument below. In a first section, we will argue that the fundamental use of the term ‘autism’ is moral and related to the *understanding* of autistic lived experience instead of a mere ‘neutral’ *explanation of* autistic difference. We do this based on the work in philosophy of science on social constructivism by Ian Hacking and one of his key sources of inspiration, Nelson Goodman. In this way we show that any theoretical explanation needs to meet the constraint of going beyond the Catch-22 articulated above. The next section will explore in detail this constraint in the light of additional elements connecting philosophical psychology as it is understood by Donald Davidson with the moral philosophy of Nomy Arpaly. In applying these elements to various essentialist and constructivist frameworks, we will contend that a reconceptualization of autism that is at odds with the current DSM-5 consensus is required to fruitfully make sense of autism (combining theoretical *and* ethical elements). The final section before the conclusion tentatively explores such a ‘theorethical’ approach based on recent insights from Bolis et al. (2017) and the Predictive Coding theory of Van de Cruys et al. (2014). We show how this conceptual space allows to express how behavior and biology are linked without either disconnecting them or reducing one to the other. Finally, in conclusion, we describe how our reconceptualization allows making good on the claims of the neurodiversity movement, without running into new moral issues and, at the same time, to create intrinsic openness to fruitful, and inherently interdisciplinary, scientific progress.

## The Fundamental Moral Uses of ‘Autism’

We start from the idea that there is no neutral use of ‘autism’ isolated from its moral use (making sense of the autistic lived experience). As such, the use of ‘autism’ (whether it’s scientific, philosophical, historical, sociological, normative, or clinical) disregarding such a fundamentally moral use can only lead to abuse. To support this claim, we first dwell on the various uses of ‘autism’ and distinguish them from what we claim to be this fundamentally *moral* use.

To do this we will not be historically exhaustive - the critical histories of the use of ‘autism’ inspiring our point are Eyal (2013) and Nadesan (2013). Instead, we highlight how the initial drive to describe prototypes [for instance clearly evident^6^ in Asperger (1944)] turned into the practice of classifying behavior as assessed in a diagnostic setting. The *direction of fit*, i.e., whether an autistic type is made to fit the people or whether the people are made to fit the type is, in our opinion, the basic difference between moral and other uses of ‘autism.’ Indeed, in *describing* autistic people, the autistic type is made to fit the people whereas in *classifying* people as autistic, a person is made to fit preconceived types. As Asperger himself noted:

*“This way, going from the expressive appearances to the core of the phenomenon consciously does without starting from a pre-given system (….) therefore, we also consciously refrain from bringing them into artificially created test situations or make them fit a stereotypical test machinery (….)”* (Asperger, 1944, p. 7, own translation).

What Asperger tried to do has a (self-acknowledged) artistic element in it, working through a process that Goodman (1978) calls *exemplification*, where knowledge of something is gained by looking at an example instantiating that something. In Goodman’s own words:

*“Exemplification, though one of the most frequent and important functions of works of art, is the least noticed and understood. (…) exemplification involves reference by what possesses to the property possessed.”* (Goodman, 1978, p. 32).

The question originally was how autistic people can be better understood as a whole, “in a dialogical relation between doctor and patient” (Ripamonti, 2016). This *understanding* is modeled on how we understand a work of art rather than on how we explain the working of a machine. As such, *exemplification describes* autistics as *prototypically* socially awkward while *explanation* requires that one *stereotypically ascribes* antisocial tendencies to every autistic person (i.e., classifies them as antisocial). We can summarize this by referring to the traditional philosophical categories of Erklären and Verstehen (as first laid down by Wilhelm Dilthey and later applied to psychiatric practice by Karl Jaspers). Table 1 provides a schematic overview of the terms used above:

**TABLE 1:** Two poles in using ‘autism.’

Erklären (explaining)	Verstehen (understanding)
Stereotypes (denotation)	Prototypes (exemplification)
Prescription (classifying)	Description (sense-making)
Scientific method (empirical studies)	Lived experience (phenomenology)

It is not our contention that the exemplifying, prototypical, use of autism is the only morally valid one, and that systematic, explanatory use based on clear empirical classification is to be avoided. The dynamic between these two poles is as inevitable as it is healthy. This is, for instance, clear from autistic self-reports taking explanatory accounts as a crucial element in their self-understanding (Hens and Langenberg, 2018). The problem only emerges when the dynamic is disturbed. On the one hand, this happens in autism research where ‘neutral’ *explanation* predominates, stripping the study object of any subjectivity, considering autistics as either biologically or socially determined to display problematic behavior. For instance, one can think of the use of animal models such as the autistic mouse or fruit fly, where behavior is merely seen as the result of a ‘gene deficit.’ On the other hand, this happens when clinical *understanding* of specific problematic cases is separated from explanation of the underlying differences autistics have in common. This approach can for instance not do justice to the common lived experience of sensory sensitivity reported across the autistic spectrum (Pellicano, 2013). One worthwhile alternative proposed is to abandon the search for one single biological explanation for the whole autistic category and instead look for specific explanations of specific elements of the over-all autistic phenotype (Happé et al., 2006; Waterhouse and Gillberg, 2014). While this recognizes autistic heterogeneity, it risks sacrificing understanding the commonality of autistic lived experiences in favor of multiple, disconnected, explanations^7^ (Chapman, 2020).

The notion of ‘autism’ naturally getting caught up in a one-sided explanatory focus is understandable, since it emerges from a medical context. As such it has been, from its very beginning, associated with problematic behavior for which a medical, therapeutic solution is sought. As stated by Ripamonti (2016), the psychiatric use of autism was instigated by Leo Kanner creating a new field of child psychiatry “to identify a narrow empirical set of criteria for medical observation and diagnosis”. In this way the ‘neutral’ explanatory question “What is autism?” (Eyal, 2013) came to dominate.

The aim of autism research in addressing this question is to uncover the mechanisms that lead to (what is deemed to be) pathological behavior. This naturally led to two competing strands of scientific explanation, depending on whether the question was interpreted within the exact sciences (looking for biological causes, the ‘reality’ behind autism) or the human sciences (looking for psychosocial factors explaining how dysfunctional autistic behavior comes about). In our view, both are morally problematic, sidestepping the primordiality of understanding the autistic lived experience by giving precedence to explanatory elements. This moral problem came to the fore when the complexity of autistic self-advocacy challenged the simple stereotypes which are the underlying assumptions of such research. For instance, Eyal (2013) describes how in the nineties self-advocacy challenged many of these stereotypes. In both cases understanding autism passes through explaining (elements of) dysfunctionality which, in line with the Catch-22, prevents autistic identification without acknowledging mental health issues and, therefore also, blocks identifying as autistic in order to prevent such mental health issues.

The new question is whether it is fruitful to try to ask the question ‘What is autism?’ in the first place. Philosopher of science Ian Hacking (1999) indeed treats autism as a paradigm example of the debate between reality and social construction, drawing his famous distinction between indifferent and interactive kinds. In the section entitled “A Dilemma” he says:

*“Here (in case of autism) we want to say both that childhood autism is (is identical to) a certain biological pathology P, and so is a “natural” kind or an indifferent kind. At the same time, we want to say childhood autism is an interactive kind, interacting with autistic children, evolving and changing as the children change*^8^ *.”* (Hacking, 1999, p. 119).

The solution as proposed by Hacking is to look beyond semantics. Instead, he proposes to look into the dynamic of *worldmaking* (Goodman, 1978), which states that worlds are made by the language we use and that we interpret the facts within these man-made – or constructed – worlds. Hacking’s concept of *dynamic nominalism*, in acknowledging that there is such an effect of language dynamically shaping reference, sidesteps the question whether (and, if so, in how far) the question “What is autism?’ allows an answer. As insightful as his analysis is (we will rely on it in section “The Looping Effect and the Attractivity of Stereotypes” below), we believe that in leaving the question open, he exacerbates his own dilemma. Indeed, the new use of autism as an illustration of a dichotomy within philosophy (of science) disregards actual autistic lived experience which, as mentioned above and as evident from the term ‘neurodiversity,’ seeks to combine an understanding of autism with an explanation of a common underlying difference. A pure social constructionist approach to autism emphasizes the link between autism and pathology, underlining our Catch-22 and denying the growing sense in the neurodiversity movement that autistics can be different without being pathological (Milton, 2017; Chapman, 2020).

Notwithstanding all this discussion, autism researchers need a working definition of autism. That definition is provided by the consensus criteria of Autism Spectrum Disorder (ASD) as spelled out in the DSM-5 (American Psychiatric Association, 2013). This consensus is a product of scientific and societal pressures (Eyal, 2013). It sidesteps the “What is autism?” debate, by a coupling of behavioral definitions (clauses A, B) to (what is broadly construed as) an innate developmental disorder (see clause C), as well as to problematic functioning (clause D). This means that autism is literally defined as autism spectrum *disorder*, implying autistic people are automatically disordered. As such, it is conceptually anchored as a pathology that can only be diagnosed if there is a manifest problem in functioning (in which case it will be both retro- and proactively understood as problematic). Although, as said above, this DSM-5 conceptualization is already challenged in autism research [Waterhouse and Gillberg (2014); Bolis et al. (2017) to name just two], such challenges have not yet led to a reconceptualization of the diagnostic practice affecting the everyday lifeworld of autistics and the practical aspects of autism research, for instance in ‘eligibility’ as autistic research participant. We come back to how our proposal constructively links to those challenges in Section “A ‘Theorethical’ View of Autism” below.

The DSM-5 conceptualization then directly leads to the Catch-22 defined in the introduction, as self-identifying as autistic entails being (literally) pathological, thereby leaving no room for understanding or making sense of non-pathological autistic lived experience^9^. That experience is complex. It includes both elements suggesting a common biological basis (as for instance in sensory sensitivity) and elements suggesting a mere mismatch with social norms (for instance a one-sided preference for typical behavior). Also, it is experienced as something that offers opportunities but *can* also cause suffering. As such, it cannot be captured by the current DSM-5 conceptualization. In the next section, we will demonstrate how the moral use of autism – going beyond this Catch-22 of autism diagnosis and research – both creates space for and puts constraints on theorizing.

## Pattern of (AB)Using ‘Autism’

In the previous section, we established that doing justice to the lived experience of autistics requires taking an ethical stance that is at odds with the current DSM-5 conceptualization of autism as necessarily pathological. The question now is to explore what kinds of constraints on theorizing follow from such an ethical stance. For this, we propose a pattern of five dimensions, created by four philosophers, which we apply to a strictly motivated classification of some illustrative autism theories^10^. It connects an ethical stalemate with a scientific stalemate through a lack of space for moral imagination and leads to a principled proposal for reconceptualizing autism to go beyond the Catch-22.

In brief, in the first element of the pattern we use Nelson Goodman’s concept of “Ways of Worldmaking” (1978) to demonstrate that the question “What is autism?” is indeed inherently world-making. Secondly, we briefly describe how unifying theories of mental phenomena, in general, are implicitly dedicated to a deep theory about brain-mind duality or identity, and as such run counter to the philosophical considerations that have been reviewed by Davidson (1970/2001). Thirdly, we demonstrate that in autism, such unifying theories create what Eyal (2013) has called “The Autism Matrix,” consolidating their social, individual and media appeal in line with the looping effect as described by Hacking (1999). In the fourth element, we demonstrate that the reductive attractiveness of these deep theoretical models reduces the space for moral imagination as described by Arpaly (2005). And finally, we demonstrate that the insistence on a deep theoretical model creates the fiction of a conceptual scheme, the “very idea of autism,” which, as Davidson (1974/2001) described for conceptual schemes in general, is unsustainable.

The pattern shows that theoretical progress is linked to a charitable everyday mutual understanding of autistic lived experience in line with the fundamental moral use of autism proposed above. With a nod to Hacking’s “looping effect of human kinds,” we call this link “the binding effect of humankind.”

To organize this section, we divide our selected illustrative autism theories in two main types. On the one hand, there are the brain (or cognitive) science theories using the methods of the exact sciences in focusing on a biological mechanism underlying autism. On the other hand, the theories that focus on the behavioral side of autism, using the methods of the human sciences to account for the emergence of (the classification of) such behavior. First, the pattern is explained in detail whilst applying it to a set of cognitive science theories. Then we apply the pattern to a selection of views on autism in the social sciences. Based on this, we conclude in Section “Reconceptualizing Autism” that avoiding moral dilemma requires reconceptualizing autism as an intrinsically ambivalent *phenomenon*, with both biological and behavioral views. Only in this conceptual space can it be expressed that behavior and nature are linked without either disconnecting them or reducing one to the other.

### Autism in the Brain Sciences

Table 2 contains a list of the cognitive science papers used in this review.

**TABLE 2:** Theories of autism in cognitive science.

Source paper	Theory identifier	Acronym used	Primary mechanism	Direction of explanation
Baron-Cohen, 2000	Theory of mind	ToM	Social	Cognitive first
Frith, 1996	Executive functioning	EF	Control	Cognition first
Happé and Frith, 2006	Weak central coherence	WCC	Attention	Cognition first
Markram and Markram, 2010	Intense world theory	IWT	Sensory	Perception first
Mottron et al., 2006	Enhanced perceptual functioning	EPF	Perception	Perception first
Van de Cruys et al., 2014	High and inflexible precision of prediction errors in autism	HIPPEA	Information processing	Perception first

This selection is not exhaustive but offers an illustrative list of cognitive science theories that have dominated debate in the scientific and autistic communities. The first three theories (ToM, EF, and WCC) are considered as somewhat outdated by many autism researchers but are still very relevant here as they are firmly established in current diagnostic practice. The latter three (EPF, IWT, and HIPPEA) have been widely discussed in the scientific literature *and* the autistic community as alternatives to the established theories. Note that the last column of the table classifies these theories as either ‘Cognition first’ or ‘Perception first’; this classification is explained in the next session when we apply our pattern to these papers.

#### The Fabrication of Facts

Goodman phrases it as follows in the section of his book on worldmaking with the same title:

*“My title, “The Fabrication of Facts,” has the virtue not only of indicating pretty clearly what I am going to discuss but also of irritating those fundamentalists who know very well that facts are found not made, that facts constitute the one and only real world, and that knowledge consists of believing the facts. These articles of faith so firmly possess most of us that, they so bind and blind us that, “fabrication of fact” has a paradoxical sound. “Fabrication” has become a synonym for “falsehood” or “fiction” as contrasted with “truth” or “fact.” Of course, we must distinguish falsehood and fiction from truth and fact; but we cannot, I am sure, do it on the ground that fiction is fabricated and fact found.”* (Goodman, 1978, p. 91).

As a starting point in our pattern, I follow Goodman in taking a closer look at the facts that are supposedly neutrally ‘found’ in cognitive science research. The fact that this is not evidently so is already clear from the review in Van de Cruys et al. (2014) classifying autism theories in two groups: “social first” and “non-social,” based on what is considered as a primary cause (see column 4 of Table 2). Autism research is therefore aware of the risk of bias in restricting the reference class of autistic people to those exhibiting the behavioral elements highlighted in DSM-5 (American Psychiatric Association, 2013, clauses A/B) to a problematic (dysfunctional) level (ibid, clause D). In terms of Goodman (1978), this risk is one of *“ordering, weighing, supplementing and deforming”* how we are making a world. In Table 2, this tension is evident from the elements in column 4, showing the autistic phenotype as, potentially at least, characterized by heterogeneity of possible primary causal factors.

We have chosen for a different classification in Table 2 last column indicated by the labels ‘Cognition first’ and ‘Perception first.’ This classification subdivides our illustrative set of theories based on the direction of explanation they take. The ‘Cognition first’ theories start from behavioral facts at a higher social or cognitive level (ToM, EF, and WCC), while the ‘Perception first’ theories (IWT, EPF, and HIPPEA) start from a variety of lower-level mechanisms. This shows that there is at least a choice in worldmaking (what is deemed essential in denoting autism) and that this choice is non-neutral^11^. It is non-neutral since this level of classification already makes a clear difference with respect to what are deemed ‘core symptoms’ and what is termed ‘comorbidities.’ Do I avert my eyes because I have difficulty reading the other’s mind (ToM) or because I see the other too vividly (IWT)? What we want to show briefly below is that the element of *exemplification* played a decisive role in the shift from ‘Cognition first’ to ‘Perception first’ theories over the last decade.

The very first brain sciences theory of autism (ToM) assumes the brain is modular and autism affects one of its modules: i.e., the social one (McGeer, 2004; McGuire and Michalko, 2011). Assuming a one-on-one correspondence between social behavior (or lack thereof) and a piece of machinery in the brain provides an immediate fit to the ‘asocial facts.’ The WCC and EF theories abandon this social-first approach but remain ‘Cognition first’ in assuming that there is a higher (typically human) cognitive faculty that is compromised in autism. The importance of this worldmaking cannot be underestimated (see also section “The Looping Effect and the Attractivity of Stereotypes” below about looping effects): they are entrenched in a matrix of worldwide diagnostic and therapeutic practices as well as in (early) autistic self-identification (Eyal, 2013). A world is made in which the autism label ties abnormal behavior to a brain deficit in a higher cognitive function^12^. The search for a unified ‘Cognition first’ explanation of autism was unsuccessful by its own lights. As Happé et al. (2006) noted the heterogeneity of autism could not be unified in this way. A suggestion was then made by Waterhouse and Gillberg (2014) to abandon the search for unified accounts in favor of looking for more ‘downstream’ explanatory accounts of *multiple* autism*s*.

The reason why the case does not end there, in cognitive science research, is directly related to the element of *exemplification* mentioned above. Indeed, prototypical reports of autistic lived experience in the form of autobiographical reports and phenomenological research [see Hens and Langenberg (2018) for both a review and an example] suggested a new research angle. The heterogeneity of the autistic phenotype at the cognitive level could be an emergent effect of the commonalities in autistic self-reports related to how the external world is perceived. Different theories are proposed under this ‘Perception first’ paradigm of which Table 2 highlights three, each focusing on a different primary perceptual element. The first (EPF) hypothesizes autism is characterized by a preference for perception of details. It focuses on a subset of autistics with ‘savant’ abilities. The second (IWT), inspired by their direct experience with their child, found support in artificial reconstruction of animal models and noting that different wiring of sensory systems had significant ‘upstream’ impacts. The last (HIPPEA) is the most recent in our list and focuses on atypical information processing leading to atypical handling of uncertainty indirectly leading to the emergence of the behavioral criteria of DSM-5 (see section “A ‘Theorethical’ View of Autism” for more detail). These theories give priority to the persistent self-reporting of perceptual elements by autistics instead of mapping the behavioral criteria to cognitive deficits. We do not want to argue that this makes them immediately correct^13^ but only suggest at this time how profound the influence from *exemplification* is with respect to world-making. In this case it even led to incorporating sensory sensitivity in the DSM-5 criteria (Pellicano, 2013).

With Goodman (1978, p. 22), we can note that at least within the biological view of autism “Comprehension and creation go on together.” The movement in recent brain science research seems to forego a direct link between abnormal behavior and brain deficits, preferring to make room for a more mundane set of facts focused on the everyday experience of and with autistics. Moving away from stereotypes and back to considering prototypes is, as we argued in section 1, essential to remain open to the complexity of the autistic lived experience^14^. In our view, it is not a coincidence that there is a move toward a notion of spectrum in DSM-5 because: “Ratings of relevance, importance, utility, value often yield hierarchies rather than dichotomies.” (Goodman, 1978, p. 10). We see the criticism noted above with respect to finding one monolithic cognitive issue to account for the heterogeneity of autism as, essentially, in the same direction. However, as already mentioned, we believe it is important to still do justice to the commonality of the autistic lived experience (see section “A ‘Theorethical’ View of Autism” for a possible convergence).

#### Comprehensive Psychological Theories

In “Mental Events” (Davidson, 1970/2001), Davidson argues for a principled restriction on psychological theories, labeling his position as that of *Anomalous Monism*. He argues that on the one hand mental events have a physical counterpart (*monism*) but that this does not mean that there can be such a thing as psychophysical laws (*anomalousness of the mental*). He dissociates his position from three alternatives, one of which (nomological monism) is the subject of this subsection (the other two positions are discussed in section “Autism in the Behavioral Sciences”). He associates the strict monist position to “the nothing-but reflex” (p. 214) stating that any mental event is “nothing but” a complex neural event (see also section “The Moral Space Afforded by Theories”).

The ‘Cognitive first’ theories, tying behavior directly to the physical outlook of the brain, are specific examples of “comprehensive closed theories” (p. 219) of cognition. Indeed, all of these theories in autism are derived from a comprehensive theory in the philosophy of mind: modularity (Fodor, 1983), leading to the pervasive metaphor of the brain as a computer (Nadesan, 2013) and combining this with evolutionary psychology into a Grand Unifying Theory (Cosmides and Tooby, 2015). The inescapable logic of this is to look for the missing link – a compromised module – and fix or cure that^15^. It seems that tying behavior directly to a neurological substrate naturally limits the room for moral imagination (see for more detail section “The Moral Space Afforded by Theories”).

The ‘Perception first’ theories are rather superficial in describing how specific neurological issues may account for the diversity of facts related to, specifically, autism. In this sense they do not suggest strict psychophysical laws relating behavior to the way brains are made up. Instead they use notions like “scaffolding” (HIPPEA) correlating behavior and brain function in a looser, statistical, way. Where ‘Cognition first’ theories talk about deficits or abnormalities *determining* behavior, ‘Perception first’ theories talk about atypicalities *affecting* behavior. We believe that this is precisely the room between the mental and the physical that Davidson argues for.

It is important to note, in conclusion of this subsection, that all the theories in Table 2 are – in the end – monistic; they try to interpret behavior based on physical elements. They are, in the terminology of (Hacking, 1999), all committed to autism *at least* also having the character of an “indifferent kind,” i.e., a kind existing independently of the looping effect discussed in the next section.

#### The Looping Effect and the Attractivity of Stereotypes

We referred above, in Section “The Fabrication of Facts,” to inescapable world-making in addressing questions like ‘What is autism?’. The follow-up question addressed in (Hacking, 1999) is how worlds so made become firmly established. This is where Hacking’s “looping effect” states that success of interactive kinds depends on how they catch on; we refer to (Eyal, 2013; Navon and Eyal, 2016) for convincing illustrations of this effect.

The media obviously promote the attractivity of a hypothesis. It helps to have slogans (or memes) ready for mass consumption. In the case of the ‘Cognition first’ theories, there are quite a number of these making the rounds, such as mindblindness, hyper-systemizing and extreme masculinity [respectively, Baron-Cohen (1997, 2002), and Baron-Cohen et al. (2009)]. The reason why such worlds achieve mass success (and thereby power the looping effect) is that they appeal to simple stereotypes. On the one hand, they allow creating stories that have an emotional appeal (Draaisma, 2009). On the other hand they link to comprehensive scientific views of science, e.g., the comprehensive psychological theory of mind-as-a-computer (Nadesan, 2013).

As Hacking notes (Hacking, 1996, p. 366), there are *“several ways in which essentialist and constructionist attitudes are not only compatible but also mutually supporting.”* As we have argued above, worthy adversaries of essentialist accounts in the mass media are constructionist accounts in which autism is “just a label” (Timimi, 2018). These two extreme positions crowd out ‘Perception first’ theories as well as the prototypical self-reporting that often inspires them. As Hacking concludes (ibid., p. 387): *“Human kinds are kinds that people want to be or not to be, not to attain some end but because the human kinds have intrinsic moral value.”* Stereotypes exculpate at the group level, whether it is because ‘it is in one’s nature’ or because ‘the environment forces us to adjust.’ In both cases they leave individuals without moral agency^16^. ‘Perception first’ theories leave the moral complexity intact but arguably become less attractive from the point of view of the looping effect as they do not deterministically generalize into something that offers blanket exemptions.

#### The Moral Space Afforded by Theories

In the previous sections, we described how the analysis of Davidson and Hacking explained the emergence and appeal of the essentialist (and social constructivist) theories of autism. In line with this analysis, Nomy Arpaly – in her paper on mental disorders *How it is not “just like diabetes”* (Arpaly, 2005) – explicitly connects the elements as touched upon above with morally problematic consequences for the people involved:

*I do not wish to argue that mental disorders aren’t like diabetes at all. Many mental disorders have all kinds of things in common with many non-mental diseases. (…) Yet, there are some significant ways in which mental disorders are not “just like diabetes,” and are like other mental states. Ignoring this can be conceptually pernicious* ***and*** *ethically risky.* (Arpaly, 2005, p. 282, our emphasis).

Indeed, moral considerations become specifically apparent in face-to-face confrontations with those to whom the psychiatric category applies. In dialog with autistic persons, it is impossible to dismiss them as ‘of another kind,’ tell them ‘it’s just a label’ (constructivist approach) or ‘it’s (not) just like diabetes.’ The analysis of (Arpaly, 2005) explicitly tries to find a middle ground (inspired just like Davidson by the Kantian notion of a moral agent as an end in itself) allowing to take into account a physical aspect of a mental disorder without dehumanizing people by reducing their behavior to that mental disorder. She does this by saying that one always has to take into account the reasons for certain behavior, no matter how disordered they may appear at first glance. In the case of autism, this means there has to be sufficient space for moral imagination in accounting for individual reactions as a genuine expression of reasons responsiveness and not merely as the reflex response to cues irrespective of their meaning. It makes all the *ethical* difference to put a melt-down by an autistic down to “not being able to cope with change” or to *imagine* it may result from the incapacity to express genuine disagreement to such a change. In what follows, we look at how the above mentioned (sets of) theories fare in this regard.

The ‘Cognition first’ hypotheses and most specifically ToM have already drawn lots of ethical controversies, especially regarding the supposed link to empathy and the credibility of autistic self-advocacy (McGeer, 2007; Milton, 2017). It looks for explanations in the smallest physical space possible. Ironically, this social theory of ASD necessarily results in the autistic person and ‘his’ social relations to the rest of the world dropping out of view. As such theories explicitly deny autistic people the basic social-cognitive abilities making up moral agency, they focus on curing autism as if it is a sophisticated kind of diabetes (Yergeau and Huebner, 2017).

“Perception first” hypotheses, on the other hand, provide ample space for moral imagination, and, see above, arguably originate from scientists exercising their moral imagination regarding what it is like to be autistic. They actively engage their readers to make sense of autistics or to wonder how it is to experience the world differently. In using terms like ‘emergence’ (EPF) or ‘scaffolding’ (HIPPEA), they relax the relation between neurology and behavior, so remaining fundamentally open to the lived experience as reported by autistic people. Also, the positives of diversity are stressed (IWT and EPF) as genuine sources of talent and potential. Autistics are seen as prototypically ‘out-of-the-box’ thinkers. At the same time ‘Perception first’ theories do not provide a deflationary account of autism as being ‘just a label’ (i.e., saying that it is *not* like diabetes at all).

“Perception first” theories then allow going beyond our Catch-22 in creating conceptual space to imagine autistic people as moral agents who are not *fully* determined by their autism, without reducing reactions of the other to “nothing but” reflexes. We turn now to how this forms a fruitful constraint on theorizing.

#### The Intellectual Space Afforded by Theories

In his article “On The Very Idea of a Conceptual Scheme” (Davidson, 1974/2001) criticizes the predecessors of the idea of radical pluralism of possible “right” world-versions that was the starting point of our pattern (Goodman, 1978). The idea that there may be multiple right versions of the world (untranslatable conceptual schemes) is untenable for him on the ground that, as far as we make sense of them, we are making sense of each other with reference to our shared world (“Principle of Charity”, p. 197). Davidson thus establishes an intimate relationship between the theoretical and the ethical. A quote from another of his papers [“Psychology as Philosophy” (Davidson, 2001)] expresses this more directly:

*“The constitutive force in the realm of behavior derives from the need to view others, nearly enough, as like ourselves. (…) The limit placed on the social sciences is set not by nature, but by us when we decide to view men as rational agents with goals and purposes, and as subject to moral evaluation.”* (p. 239, our emphasis).

In this light, let us look at our sets of cognitive science theories and their space for scientific creativity and fertility. We have already pointed to the fact that ‘Perception first’ hypotheses seem to talk more both to and from moral imagination. They also tend to start from familiar facts rather than from a set of high level social or cognitive concepts that are unavoidably linked with social norms and subjective assessments from the neurotypical standard. As Milton (2017) coins it, the tendency to start from here can be seen as a ‘Double Empathy Problem’ where the charge of social-cognitive deficits actually is a sign of a lack of empathy or understanding by the neurotypical world of neurodiverse ‘forms of life^17^’ (Chapman, 2019).

Once a choice is made to commit oneself to specific mechanistic causal chains (nomological monism), the object of study is stripped from the relevance of its subjective, lived experience. The result is that one scientific discipline, in this case, the brain sciences as an extension of the exact sciences, also becomes a master to the others. The ethical stalemate of a Catch-22 can’t be avoided as autistics are ‘locked in’ in their dysfunction. It also goes together with a scientific stalemate as the insights from other scientific disciplines are barred from contributing to a full dynamic of *explanation for better understanding*. We have seen that “Perception first” theories, insofar they are compatible with anomalous monism, allow to take into account ‘what it is like to be autistic?’ (and arguably a theory like HIPPEA starts from this very question). We explore in Section 4 how such hypotheses can productively combine with other disciplines in what we will call a ‘theorethical stance.’ For now, it is important to note that such a stance does not commit us to a relativistic notion of for instance autism, this is the way Davidson puts it:

*“Of course, truth of sentences remains relative to a language, but that is as objective as can be. In giving up the dualism of scheme and world, we do not give up the world, but re-establish unmediated touch with the familiar objects whose antics make our sentences and opinions true or false.”* (Davidson, 1974/2001, p. 198).

According to Davidson, we must give up the idea of deep, conclusive theories to accept that we are continuously in discussion with each other trying to adapt our language to how the world is and how we can improve our mutual everyday understanding (of the world *and* each other). It is in this sense that we believe that the concepts of moral and intellectual space are tightly related. A deep theory is an answer to “What is autism?” where individuals are made to fit a theory. This closes in the same stroke the room for understanding individual experiences and creating new explanations (or worlds) in which such experiences *can* be understood.

### Autism in the Behavioral Sciences

Above we reviewed the brain sciences use of autism. We have described how “Cognition first’ theories are incompatible with our pattern. In this subsection, we apply this pattern to autism research in the behavioral sciences. The comparative brevity of the discussion is due to two reasons. First, having established the pattern, it’s more readily applied. Second, given the disparate nature of theories in these fields, we have limited ourselves to selecting two theories. These theories are selected not because they are fully representative for their fields but because they allow us to best illustrate the conceptual force of our pattern. That said, we do believe that the specific theories selected are relevant independent of our argument as they had a significant impact on contemporary thinking about autism^18^.

The selection of autism research papers discussed in this section is given in Table 3.

**TABLE 3:** A sample of human sciences autism research theorizing.

Source	Theory	Primary mechanism	Type of dualism
Hobson, 2011	Developmental psychology	Joint attention	Nomological
Verhoeff, 2015, 2013	(De)construction	Dysfunctionality	Anomalous

The first column of Table 3 identifies books or papers used as a source. The second column shows an identifier of the human sciences approach taken. The third column (parallel to the explanatory mechanism used in Table 2) states the mechanism taken as primary in accounting for autism and column four contains a categorization of the theory in light of a central element of our pattern. It is the latter element that drives us to take the two theories as the relevant ones. Indeed, in Davidson (1970/2001, p. 213), the fourfold classification of *“theories of relations between mental and physical events”* is given. We described in Section “Comprehensive Psychological Theories” the difference between nomological and anomalous monism establishing a principled preference for the latter. The two others are nomological and anomalous dualism, the views that there are, respectively, purely psychological laws which need not have a parallel in the physical or that there are no “laws correlating the mental and the physical.” Given that we argued that anomalous monism is, in the context of our pattern, a requirement to avoid the moral dilemma originally sketched, our work in this subsection is for the most part critical. This doesn’t mean that scientific accounts starting from behavior have no contribution to make. It just means that any behavioral sciences theory (just as any cognitive sciences theory) not committed to interdisciplinarity leads to the same kind of combination of moral dilemma and scientific stalemate that we established above for ‘Cognitive first’ theories (and for other theories committed to nomological monism).

#### Joint Attention

The world made by Hobson (2011) is one of psychological regularities between the facts of child-rearing and the resulting autistic behavior. Although it isn’t denied that there may *also* be facts of nature in play (reference is made to ToM but also to blind and deaf people), the law leading to problematic autistic behavior is squarely based in nurture and is thus purely psychological. This leads to discarding some elements of the autistic phenotype prominent in autistic self-reports as described above (most notably sensory sensitivities). Indeed, by questioning the sense of autistic self, see (Constant et al., 2018), the mere validity of autistic self-reporting is questioned.

Hobson (p. 151 ff.) proposes a comprehensive psychological doctrine by making a selection of facts based on the insights of *attachment theory* with focus on joint attention. This theory holds that adult behavior can be characterized in several types (secure, insecure) and such characterization can be reduced to the quality of the attachment between the child and the parent during the rearing phase. The theory establishes a law between abnormal nurture and abnormal behavior. As such, it confirms and entrenches stereotypes of (asocial) autistic behavior in a simple paradigm that can be easily communicated. In a sense, autism is a deviation that is accounted for simply by reference to a deviant child-rearing situation. Autism is used to pinpoint how nurture should be normalized to avoid deviancy. Such a view is compatible, literally through parallelism^19^, with physicalist assumptions as mentioned above.

Although such a law is far removed from the concept of autism as a disease like diabetes, it is easy to see how it is similarly destructive of moral imagination. Indeed, the abnormal behavior concerning social and communicative norms is “nothing but” the inevitable outcome of how one was raised in childhood. In our example of coping with change (see section “The Moral Space Afforded by Theories”), the autistic meltdown is explained by the earlier attachment instead of by the content of change reacted to. This to the extent that it is questioned whether autistics are not just like robots going through the motions (Hobson, 2011), locked up in idiosyncratic conceptual schemes. The category of autism is claimed for developmental psychology (and more specifically for psychoanalysis). Although Hobson abandons a simple biological-mechanistic explanation (based on genes or brain function) in favor of an interactional approach, it does not seem to surpass a mere causal-explanatory approach (a point also made by Karl Jaspers when criticizing psychoanalysis as reductionistic).

#### (De)Construction

This account would not be complete if it did not address head-on the tension in the pattern we propose. Indeed, a pluralist position as per Goodman (1978) shares the anomalousness of the mental with Davidson’s preferred scheme of anomalous monism. There is, however, a crucial difference: whereas the latter allows some relevance to the question ‘What is autism?’, the former – when taken to the extreme – seems to deny just that^20^.

The analysis of (Hacking, 1996, 1999) indeed already points to an (at least partly) purely social dynamic of constructing our understanding of reality. The sociological analysis of (Eyal, 2013; Navon and Eyal, 2016) traces the anomalousness of “autism” to a matrix of people sustaining and changing an essentially historical understanding of autism^21^. Whereas these analyses are themselves non-committal (see section “The Fundamental Moral Uses of ‘Autism’ ”) on the relevance of asking “What is autism?”, they resonate in this deconstructionist position denying relevance to the “What is autism?” question.

To illustrate this, we take the two papers by Verhoeff (2013, 2015) arguing from the historicity and the lack of demarcation of the concept of autism to the conclusion:

*I suggest that basic autism research should focus on experiences of impairment and distress, and on how these experiences relate to particular (autistic) behaviors in particular circumstances, (…)* (Verhoeff, 2013, p. 443).

This particularistic conclusion is explained in Verhoeff (2015, p. 442), arguing against the *“supposed historical continuity in the meaning of autism.”* What we are left with is the image of boundless looping of a concept unanchored in any reality in the aboriginal world. Such a situation leads to a deflationary account of autism, inspiring us to look at the case particulars and be skeptical of the categories that are reified behind them.

For Verhoeff, the primary use of autism is the clinical, psychiatric use where the label of autism happens to help co-ordinate therapeutic practice – but where we should suspend judgment on any reality behind it as the priority is to *treat* the case. Although the presentation of autism as an inherently flexible and dynamic concept opens up the possibility of many different ways of autistic selfhood, it also risks trapping autism purely in psychiatric practice and may deny autistics a transhistorical understanding for their way of being (Ripamonti, 2016). The idea of boundless looping, in so far as it incorporates an idea of radically incomparable conceptual schemes risks denying autistics the very thing so evidently looked for by them in the movement of neurodiversity: a sense of community based on a neurological difference allowing people to identify as autistic without necessarily being dysfunctional or pathological. (De)construction highlights “the looping effects of human kinds” while remaining blind to the “binding effects of humankind” per the “Principle of Charity” (Davidson, 1974/2001). In this way it establishes a deep theory about the historicity of *understanding* autism and is blind to the moral impetus of trying to *explain* a timeless reality of autism as a diverse way of being (Arpaly, 2005) sharing everything that is human (Davidson, 1974/2001).

With this, we have connected our detailed argumentation per our pattern to the hypothesis expressed at the end of Section “The Fundamental Moral Uses of ‘Autism’ ”: we need explanations of autism *to* better understand autistics [in fact, see Milton (2017): to better understand each other]. Avoiding the Catch-22 – avoiding moral dilemma – de facto puts ethical constraints on theorizing. In line with the idea of Anomalous Monism it also de facto requires interdisciplinary cooperation across brain and social sciences without either claiming to be primordial. The question we turn to now is how to construct a conceptual space wherein we can theorize without violating the ethical constraint.

### Reconceptualizing Autism

So, if we refuse to accept that in the case of autism we should not: “*On the level of theory, (…) flit back and forth between extremes as blithely as a physicist between particle and field theories”* (Goodman, 1978 p. 119), then what to do? The lead we may follow is suggested by Goodman (p. 57): “*A reconceptualization of the problem (…) may help to clarify (…) moot matters.”* More specifically, he says (p. 68) “*Symptoms, after all, are but clues; the patient may have the symptoms without the disease, or the disease without the symptoms.”*

What we have shown is that the current conceptualization of autism as per DSM-5 allows theorists to flit back and forth between nomological theories that either take psychosocial (see section “Joint Attention”) or biological (see section “Autism in the Brain Sciences,” ‘Cognition first’) elements as fully determining “What is autism?”. Similarly, such a DSM-5 conceptualization allows flitting back and forth between seeing autism either through the lens of the brain sciences (nature, see section “Autism in the Brain Sciences”) or through the lens of behavioral sciences (nurture, see section “Autism in the Behavioral Sciences”). In the latter case, one can reject the question “What is autism?” as irrelevant as only the problematic behavior counts from a perspective of the clinic, and that is ‘social construction through-and-through.’ While each of these positions have a certain appeal, they all run into the ethical stalemate exemplified by a Catch-22 that is borne from autistic lived experience. We have also shown that this ethical stalemate leads to scientific stalemate where different scientific disciplines claim precedence over the others, and where one is unable to do justice to the moral demand for an explanation of autism to achieve a better understanding of autistics.

This latter demand is we believe the central one in the neurodiversity movement which, as we noted in the introduction, is intuitively attracted to (as the term itself suggests) a combination of elements of both indifferent and interactive kinds. It seems the only way to capture this is to move to a reconceptualization compatible with Anomalous Monism where it can be expressed that objective neurological differences are non-deterministically linked to (what is now deemed to be) problematic behavior without such difference limiting the moral imagination to interpret autistic reactions as “nothing but” the expression of such a difference.

This situation is like that of quantum physics where quantum phenomena can only be described by combining both particle and wave descriptions but where the latter descriptions are mutually exclusive, i.e., irreducible to each other (Bohr, 1955, 1960). In the case of autism we then need to independently conceptualize “autistic nature” (the individual “particle” view of autism as is studied in the brain sciences) and “autistic behavior” (the interactional “wave” view of autism as is studied in the social sciences). Any explanation of autism combining these two elements will then automatically leave some indeterminacy. This indeterminacy does direct justice to the moral agency of autistics, in other words they are not (physically or socially) determined to get confronted with mental health issues. That said, in keeping with the Double Empathy Problem (Milton, 2017), whether or not they *can* enduringly cope with these situations depends as well on the understanding of neurotypical others. It also means such explanations will, necessarily, be interdisciplinary as, in keeping with Anomalous Monism, they intrinsically need to combine elements from the exact and social sciences. This can be illustrated with a simple example from disability studies: social participation is a challenge for people in wheelchairs, but this can only be understood in a specific case by combining inaccessibility of locations for wheelchairs with somatic reasons for requiring a wheelchair. There is no necessity to see a challenge with respect to social participation as a dysfunction intrinsic to the way the individual is. The dysfunction is, in fact, only due to the measures for accessibility being insufficient (Glackin, 2010; Chapman, 2020).

What we have shown is that if we abandon the DSM-5 conceptualization where the definition of autism is anchored in dysfunction, we have a principled way forward to express that behavior and biology are linked without either disconnecting them or reducing the one to the other. In line with our pattern, such a reconceptualization not only avoids the moral dilemma inherent in our Catch-22 but also links to the trend in autism research to work in multi-causal frameworks that allow for interdisciplinary contributions without giving priority of any of the disciplines (Bolis et al., 2017). Specifically, it shows it is crucial to integrate quantitative *and* qualitative research into the lived experience of autistics (Hens and Langenberg, 2018). What remains is to show *how* such integration can be done, to this we turn in the next section.

## A ‘Theorethical’ View of Autism

In this section, we want to show how an account developed in a common space for the ethical and theoretical – in short a ‘theorethical’ approach – opens up the perspective of more fertile interdisciplinary theorizing. In this paper, we cannot obviously elaborate such a theory; we can only attempt, based on current autism research, to illustrate how starting from the desire to *understand* autistic lived experience produces *explanations* productively tying insights in behavioral and brain sciences (“wave” and “particle” views of autism) together. In working out such an illustration we will be able to indicate in our conclusion how a reconceptualization as we propose can be implemented with respect to the current DSM-5 conceptualization in a way allowing cooperation between researchers to work out a testable ‘theorethical’ approach.

A first element is to see human behavior as emerging from intersubjective processes. This is pursued by researchers in the enactive tradition, notably (Fuchs and De Jaegher, 2009) and (Di Paolo and De Jaegher, 2012). The concept they elaborate is that of participatory sense-making (Fuchs and De Jaegher, 2009; De Jaegher, 2013) in which individuals constantly try to make sense of each other and psychiatrically problematic behavior results from the breakdown of that intersubjectivity. Such a breakdown then becomes – as per our framework in the above section – predictable but also preventable in conceptualizing its multi-causal background. Bolis et al. (2017) make this more explicit in their Dialectical Misattunement Hypothesis (DMH)^22^ rethinking *“psychiatric conditions as disorders of social interaction.”*^23^ Their hypothesis is aligned with ours, as is evident from this quote: *“To put it simple: intra-subjective parameters are deployed for capturing individual mechanisms, while intersubjective ones describe potential emergent processes on the collective level.”*

Here we have in embryonic form a ‘Schrödinger equation’ for autism where the intrasubjective parameters relate to the ‘individual particle’ view of autism as researched in the exact sciences and the intersubjective parameters to the ‘interactional wave’ researched in the social sciences. They come together in the autistic individuals whose lived experience reports are therefore, in line with our argument, crucial in order to ‘make (participatory) sense’ of how explanations of autistic nature and autistic behavior interrelate in an indeterminate way which remains open to the individual moral agency of autistic (and non-autistic!) individuals.

The remaining issue now is to define autistic nature and autistic behavior independently. To do this we take a closer look at a possible hypothesis of autistic nature that is in line with dialectical misattunement as stated by Bolis et al. (2017). Their DMH is articulated relying on a Predictive Coding (PC) framework. One PC theory of autism, “High and Inflexible Precision of Prediction Error in Autism” or in short HIPPEA (Van de Cruys et al., 2014) is mentioned above as one of the ‘Perception first’ theories in Section “Autism in the Brain Sciences.” In HIPPEA it is hypothesized autism consists in a specific way of dealing with perceptual uncertainty. As the PC framework is a computational framework, autistic nature, following HIPPEA, is an atypical way of processing the difference (the “Prediction Error”) between perceptual input and prior expectations. Autistic nature then consists in atypically giving a High and Inflexible weight to the difference between both (i.e., in an atypical importance of “Precision”). This then leads to a learning style at odds with the requirements of a typical environment which, via the notion of scaffolding mentioned higher, leads to development of behavioral traits associated to autism – for a developed example of this process related to the atypical development of a self, see a paper we co-authored: (Constant et al., 2018).

Now, if we would be able to measure such atypical precision independently of a full behavioral diagnosis including dysfunction (as is the goal of such research programs) then we can picture a situation in which dysfunction (or even misattunement) is no longer a necessary aspect of the conceptualization of autism. To be clear: this measurement would *not* exhaust the meaning of autism (that would bring us back to the fully reductionist nomological monism!). It would just be the cognitive science element which can be pursued somewhat independently from the social sciences research but always with the realization that both research efforts are firmly anchored in the commonalities of an autistic lived experience that no longer needs to be dysfunctional.

The latter sentence indicates that the resulting conceptualization of autism will be as dynamic as the history of defining autism was and, as we contend, as autistics *are*. This should not lead to despair because, as our pattern shows, it is precisely this dynamic that allows us to get to explaining *all* the facts allowing to improve self-understanding of autistics as well as mutual understanding between neurodiverse and neurotypical people (as much as possible avoiding misattunement).

There is, by the way, independent evidence that such a dynamic understanding is indeed what recent autism research points toward. Johnson (2017) makes a step of relying on multi-causal models *entangling* behavior and neurobiology via ‘common developmental pathways.’ Such a view is moreover compatible with findings of Waterhouse and Gillberg (2014) mentioned above. Indeed, multiple discrete neurobiological or genetic causes can, for instance, converge to some extent in such developmental pathways. This could account for both a commonality of autistic experience *and* a certain level of heterogeneity in how certain subgroups specifically present an endophenotype. We suggest that it would be worthwhile, based on this reconceptualization, to work out how an epigenetic account, for instance as proposed by Jablonka and Noble (2019, specifically Figure 1), could constructively contribute to a dynamic understanding of the *phenomenon* autism. Maybe it helps in closing to refer to the *phenomenon* of ‘being a good basketball player’: we know a certain biological predisposition helps but at the same time we know that there are many ways to achieve this behavior based on the plasticity of our biology and the canalization of our talent.

The point of this example is to show that openness to understanding autistic lived experience is crucial to achieving truly interdisciplinary breakthroughs allowing to uncover pathways in which ‘particle’ and ‘wave’ explanations get entangled. Unfortunately – and despite efforts of autistic (and many non-autistic) people – this culminated too often in mental breakdown (and what was deemed dysfunctionality) but the latter is not necessary. What *is* necessary is to break the one-to-one association between autism and pathology. To this we turn in our conclusion.

## Conclusion: Introducing ‘Autism Related Disorder’

This has been a long and complex argument leading to an intuitive conclusion at the center of the neurodiversity movement: neurological diversity is a fact that should not be identified with psychiatric problems. Even stronger than this, autism should not even be *defined* with reference to behavior that is deemed dysfunctional. We have illustrated how such ‘theorethical’ approach might look like. This obviously does not mean that the correct interdisciplinary approach will look like this. What it *does* mean, however, is that a strict identification of autism with an ASD DSM-5 diagnosis needs to be abandoned. We propose, in line with the reconceptualization that we proposed, to rename it to Autism Related Disorder (ARD). Doing so is a pragmatic way to sever the link between autism and pathology whilst at the same time acknowledging that given circumstances, many autistics experience breakdowns of intersubjectivity that require a formal diagnosis to get access to the care they need (Glackin, 2010; Mole, 2017). This not only liberates autistics from psychiatric stigma but, in one and the same move, liberates scientists to fruitfully cooperate beyond disciplinary boundaries integrating knowledge from various strands of brain and behavioral sciences, always anchored in autistic lived experience. In a pragmatic spirit this can be done keeping the DSM-5 criteria identical awaiting new insights of autism research. It will be up to the research community to agree on criteria unrelated to dysfunctionality in order to circumscribe who is autistic. Inspiration can be drawn from the autistic community itself as the gatekeeping function of a formal diagnosis is, by and large, absent from the neurodiversity movement. It anyway also aligns with research practices working with subclinical autism traits (Waterhouse and Gillberg, 2014).

We have argued that to go beyond the Catch-22 of autism research and diagnosis we are to consider all the moral implications of (not) asking ‘What is autism?’. The structure of our argument is a tight fit between theoretical and ethical elements. It is impossible to move beyond the stalemate between accounts of autism in different scientific disciplines if it were not for the pressure of autistic (self-)advocacy. “What is?” questions, when inspired by the motivation of understanding lived experiences, need not be avoided and certainly not discarded. We just have to work with a more dynamic and flexible understanding of autism that allows to develop new ideas in which disciplines can, again, cooperate on fertile new ground. The foundation of this can be, as we suggested, simply obtained by definitively breaking the link between autism and pathology via renaming ASD to ARD in a newly revised version of the DSM.

### Postscriptum

The reviewers, quite rightly, point out that our argument leaves the question ‘What is autism?’ maybe more unsettled than it was per the current status quo. We have not tried to preempt the empirical findings related to autism via a purely armchair analysis. Rather, we believe it is of importance to unsettle the status quo precisely in order to allow empirical analysis that may do justice to a commonality of autistic lived experience in all its dynamism and heterogeneity. We do not pretend that our ARD proposal is a magic solution (or in fact can be properly understood outside of the nuance of our argument). We do believe that, in the present context, it best honors the fact that autism is a unique combination of sensitivities which may lead, in the same person, to disability *and* to exceptionality. In this vein, we refer to the work of Elizabeth Barnes (2016) *The Minority Body* which decouples disability from the negative connotation of disorder and - in its stead – focuses on ‘disabling conditions’ experienced by ‘minority bodies.’ In this sense, we explicitly propose to view autism as one specific type of a ‘minority brain.’ This obviously leaves empirical research with a new challenge: how to ‘define’ an autistic population vis à vis the (typical) test population. Here we want to remark that this challenge is not entirely new in autism research where use is already often made of notions like ‘subclinical’ traits and where measures other than formal ASD DSM-5 diagnosis are regularly used. We do not believe that there is a simple solution to this problem like, for instance, relying on self-identification (which can be perfectly fine outside of empirical research). Indeed, such self-identification will always depend on personal choice and societal circumstance and will, hence, be powerless to close in on common sensory sensitivities reported across the autistic spectrum. We believe that it is of importance to embrace this challenge rather than to try to find an armchair shortcut around it, precisely because this will be the only way to recognize the dynamism embedded in – what we have argued – is the key touchstone of autistic lived experience.

## Author Contributions

JB: initial proposal and manuscript and final revisions. KH: initial manuscript and intermediate revisions. Both authors contributed to the article and approved the submitted version.

## Conflict of Interest

The authors declare that the research was conducted in the absence of any commercial or financial relationships that could be construed as a potential conflict of interest.

## Footnotes

1 The corresponding author of this paper has been diagnosed with ASD as an adult. It was his choice to use the term ‘autistic’ throughout this paper, in line with autistic preferences, cfr. Kenny et al. (2016). We also use the terms autism and Autism Spectrum Disorder interchangeably without semantic concessions such as “Autism Spectrum Condition” (ASC). While we applaud the sensitivity shown by those using ASC, this concession does not go far enough to avoid the issues raised in this paper.

2 We refer to the video “In My Language” of Amanda Baggs (a non-verbal autistic person) criticizing, inter alia, a distinction between HFA and LFA: https://www.youtube.com/watch?v=JnylM1hI2jc.

3 As said at the end of the first paragraph, we do not imply that no fruitful attempts in this direction are taken. In fact, we build on such attempts at the end of the paper to formulate our conclusion. We want to acknowledge the reviewers for pointing out a continuity between this trend in research and in medical practice and our argument in this paper.

4 For reference:(A) Persistent deficits in social communication and social interaction across multiple contexts, as manifested by the following, currently or by history,(B)  Restricted, repetitive patterns of behavior, interests, or activities, as manifested by at least two of the following, currently or by history,(C)  Symptoms must be present in the early developmental period (but may not become fully manifest until social demands exceed limited capacities, or may be masked by learned strategies in later life), and,(D)  Symptoms cause clinically significant impairment in social, occupational, or other important areas of current functioning.

5 Such a potential is partly recognized in the World Health Organization’s International Classification of Functioning, Disability and Health for ASD (Boölte et al., 2014) where provision is made to score aspects of function either below or above a typical level of functioning (we thank one of our reviewers for pointing this out to us). Still, this nuance is made within a conceptualization of autism *as a* disorder.

6 While writing this, the supposition of collaboration of Asperger with the Nazi regime was confirmed. As horrific as this is, Czech (2018) states: “Regarding Asperger’s contributions to autism research, there is no evidence to consider them tainted by his problematic role during National Socialism.”

7 We are grateful to an reviewer for pointing out this alternative (explored in recent autism research). We will come back in section 4 to how this alternative can be made compatible with common autistic lived experience after a proper reconceptualization of the diagnostic approach in DSM-5.

8 By this time, the biological (biomedical) view dominated explanatory debates. However, as we mentioned, and will argue in more detail later, psychosocial explanations share the same lawlike (nomological) explanatory type of approach wherein autism is reduced to a mechanism, albeit in this case one where autistic behavior will be determined primordially by developmental psychology given certain social child rearing conditions.

9 That it was never intended in this way can be seen from a remark by Asperger (1944) that autism vs. present “inside and outside of the hospital.” We remark in passing that changing *Disorder* to *Condition*, moving from ASD to ASC, does – in our view – not sufficiently break the connection with dysfunction. We will, however, not argue this specific point and simply use ‘autism’ as most widely accepted term.

10 This should not be read as a review paper. The autism theories in this section are selected in order to bring out the pattern linking ethical and scientific stalemate. Although they are illustrative of the type of theories that have most animated debate in the scientific and autistic communities, we do not claim they fairly represent recent autism research. In fact, in Section “A ‘Theorethical’ View of Autism” we address constructively how some recent research displays a trend that is, albeit implicitly, aligned with the proposed pattern.

11 In social construction, this element is viewed to be skeptical of any physical explanation of autism [see Hacking (1999); Eyal (2013) and Verhoeff (2013, 2015)]; the patterned review of such views will be further discussed in Section “Autism in the Behavioral Sciences” below.

12 It is significant that in line with the ‘Cognition first’ theories reviewed that there was significant literature on why autistic self-reporting could not be relied on because of lack of self-knowledge [see reaction of McGeer (2004) on Frith and Happé (1999) and for a review and alternative: (Constant et al., 2018)].

13 In fact, we believe that the EPF-hypothesis with its insistence on the ‘savant’ element is too restrictive to do justice to the commonality in autistic lived experience. The IWT-hypothesis puts all emphasis on animal and brain models and in so doing remains closed to the social elements influencing autism.

14 A movement that, in the words of Goodman (1978, p. 34), can be seen as a move back to a style (or personality) as a “complex characterization that serves somewhat as an individual or group signature (…),” clearly reminiscent of the initial attitude of (Asperger, 1944) as reviewed above.

15 Note that Fodor himself in (Fodor, 1983) explicitly argued against the possibility of a (central) executive module supposedly compromised in the theory of EF (see p. 101). Also, as mentioned at the end of the previous paragraph, this does not mean that autism theories relying on brain imaging techniques need be based on this simple computer metaphor and may be helpful in mapping out more complex relations between brain activity and behavior (we are grateful to a reviewer for pointing this out).

16 This is a criticism that can in our view also be leveled against some neurodiversity views. The view that is expressed in this paper introduces a crucial caveat to such views: there is always some room for a coming-together of minds across the neurological spectrum and this room requires that both sides are sensitive to the limits and constraints of the specific other in front of them (Milton, 2017).

17 We mention in passing that the structure of Milton’s argument is, in our view non-coincidentally, very similar in the specific field of autism to the one from Davidson we leverage here. There is also a clear parallel with the concept of participatory sense-making of De Jaegher (2013) that we will use in Section “A ‘Theorethical’ View of Autism” to make the bridge with interdisciplinary approaches as for instance suggested by Bolis et al. (2017).

18 We thank our reviewers for pointing us to the necessity of this disclaimer. Specifically for the field of developmental psychology interested readers would find it useful to consider the work of V. Reddy for a full, balanced treatment of attachment theory.

19 Parallelism is one of the accounts classified by Davidson under Nomological Dualism.

20 On p. 100 of (Goodman, 1978), he says that we have to: “give up our futile search for the aboriginal world” (ibid., p.100). For Davidson, see higher, such an “aboriginal” world is our starting point, from which the very idea of pluralist conceptual schemes is derived.

21 Although not further explored here, it is worth noting that – in line with Hackings view of the intimate connection between deconstruction, unmasking and social construction – that a similar analysis of the deconstruction approach common in disability studies (Goodley and McGuire) should be conducted. In these approaches as well there is much commonality in starting point with our analysis but an incompatibility in ultimately claiming that the social sciences are to have the final say.

22 I am indebted to Axel Constant-Pruvost for pointing out the proximity of this hypothesis with the ideas pursued in this paper.

23 Put more elaborately for the case of autism: there is an “autistic nature” that, when not appropriately taken into account, in social settings is prone to lead to an intersubjective breakdown associated with what is defined as dysfunctional “autistic behavior” in the sense of DSM-5.
